# Postpartum Treatment for Substance Use Disorder Among Mothers of Infants with Neonatal Abstinence Syndrome and Prenatal Substance Exposure

**DOI:** 10.1089/whr.2020.0128

**Published:** 2021-06-01

**Authors:** Laura J. Faherty, Sara Heins, Ashley M. Kranz, Bradley D. Stein

**Affiliations:** ^1^ RAND Corporation, Boston, Massachusetts, USA.; ^2^ School of Medicine, Boston University, Boston, Massachusetts, USA.; ^3^ RAND Corporation, Pittsburgh, Pennsylvania, USA.; ^4^ RAND Corporation, Arlington, Virginia, USA.; ^5^ School of Medicine, University of Pittsburgh, Pittsburgh, Pennsylvania, USA.

**Keywords:** Medicaid, neonatal abstinence syndrome, opioid use disorder, postpartum, pregnancy, substance use disorder

## Abstract

***Background:*** Little is known about rates of substance use disorder (SUD) treatment for women in dyads affected by substance use in the immediate postpartum period. This study's objectives were to (1) identify characteristics of mothers of infants with neonatal abstinence syndrome (NAS) and/or prenatal substance exposure (PSE) who did or did not receive SUD treatment in the first 60 days postpartum and (2) describe timing of treatment receipt.

***Methods:*** This descriptive study examined linked mother–infant dyads using Medicaid data from Louisiana, Massachusetts, and Wisconsin for 2006–2009. Dyads were included if the infant had NAS and/or PSE. Descriptive statistics on sociodemographic characteristics, prenatal SUD, mental health conditions, Medicaid enrollment, and health care utilization were reported for women who did and did not receive SUD treatment in the first 60 days postpartum. The distribution of each variable was compared using chi-square tests. The timing of first postpartum treatment in weeks since delivery was examined.

***Results:*** Among Medicaid-insured women whose infants had *in utero* substance exposure, 15% received any postpartum SUD treatment. Fewer than half were diagnosed with SUD prenatally. Of those who received postpartum SUD treatment, 68% had received prenatal treatment. No association was observed between postpartum SUD treatment receipt and months of Medicaid enrollment in the year before delivery, prenatal visits, or postpartum visit attendance.

***Conclusions:*** Most women who likely need postpartum SUD treatment did not receive it and multipronged solutions are needed. These findings provide a useful baseline for evaluations of policies aimed at improving maternal health.

## Introduction

Substance use among women of reproductive age is a complex public health challenge. From 1999 to 2014, rates of opioid use disorder (OUD) diagnosed at the time of delivery more than quadrupled in the United States,^1^ and the incidence of neonatal abstinence syndrome (NAS) due to prenatal opioid exposure increased sevenfold.^2,3^

The postpartum period is a vulnerable time in a woman's life-course, particularly for those with substance use disorders (SUDs). Only about half of women insured by Medicaid attend their postpartum follow-up appointment, recommended to occur about 4 to 6 weeks after delivery.^4^ Women with SUDs are particularly in need of engagement in care in the postpartum period given their increased risk of adverse obstetrical outcomes^5^ and of overdose in the months following delivery.^6^ However, treatment access can be difficult for women with SUD whose care is fragmented across multiple systems, who struggle to find SUD treatment options that provide childcare services or permit children to stay with them in residential settings, and for whom state policies often terminate Medicaid coverage 60 days after delivery for those whose eligibility resulted from pregnancy.^7^

Mothers with SUD whose infants are diagnosed with NAS or prenatal substance exposure (PSE) in the neonatal period are a readily identified high-risk population that should receive evidence-based postpartum SUD treatment, whether newly initiated or continuing from the prenatal period. While many SUD treatment barriers for pregnant women have been described^8,9^ and a prior study has examined treatment receipt among women with SUD and OUD in the 24 months surrounding delivery,^10^ little is known about rates of SUD treatment for women in dyads affected by substance use in the immediate postpartum period (*i.e.*, the start of the so-called fourth trimester)^11^ or the sociodemographic and clinical characteristics associated with treatment receipt. Therefore, using an innovative but infrequently used approach of linking mother–infant dyads in Medicaid data,^10,12^ we sought to (1) identify sociodemographic, health insurance, utilization, and clinical characteristics of mothers of infants with NAS and PSE who did or did not receive SUD treatment in the first 60 days postpartum, and (2) for those receiving treatment, describe the timing of postpartum treatment receipt. Building on literature describing factors associated with prenatal SUD treatment receipt,^13,14^ we hypothesized that most mothers in substance-affected dyads would not receive timely postpartum treatment for SUD, and that race, urbanicity, duration of Medicaid enrollment, prenatal care attendance, and co-occurring mental health conditions would be associated with receipt of SUD treatment in the 60 days after delivery.

## Methods

### Study design and data sources

Medicaid Analytic Extract (MAX) data files were used for this descriptive study. To construct mother–infant dyads, we linked two samples of MAX data. The infant sample used 2006–2009 data obtained for a study of early childhood dental care utilization^15^ and included all Medicaid-enrolled children younger than 6 years of age from selected states who received dental services in medical offices and a stratified random sample of Medicaid-enrolled children younger than 6 years who never received such services. Children who received dental services received a weight of one and controls received a weight equal to the inverse proportion of controls sampled (*e.g.*, if 20% of children who did not receive dental services were randomly selected in a given state, those children each received a weight of five to account for other children that were not sampled).^16–18^ This sample was weighted to be representative of all Medicaid-enrolled children younger than 6 years of age in these states and years. The maternal sample used 2005–2009 data obtained for a study of pharmacotherapy for OUD^19,20^ and included all Medicaid-eligible women in the states and years of the study. Maternal data from 2005 were included to provide information on the prenatal period for deliveries occurring in 2006. Three states—Louisiana, Massachusetts, and Wisconsin—were selected for this analysis based on overlapping data availability in both the maternal and infant samples and high linkage rates from a prior study constructing mother–infant dyads using MAX data.^21^

### Sample construction

**Appendix FIG. A1.**
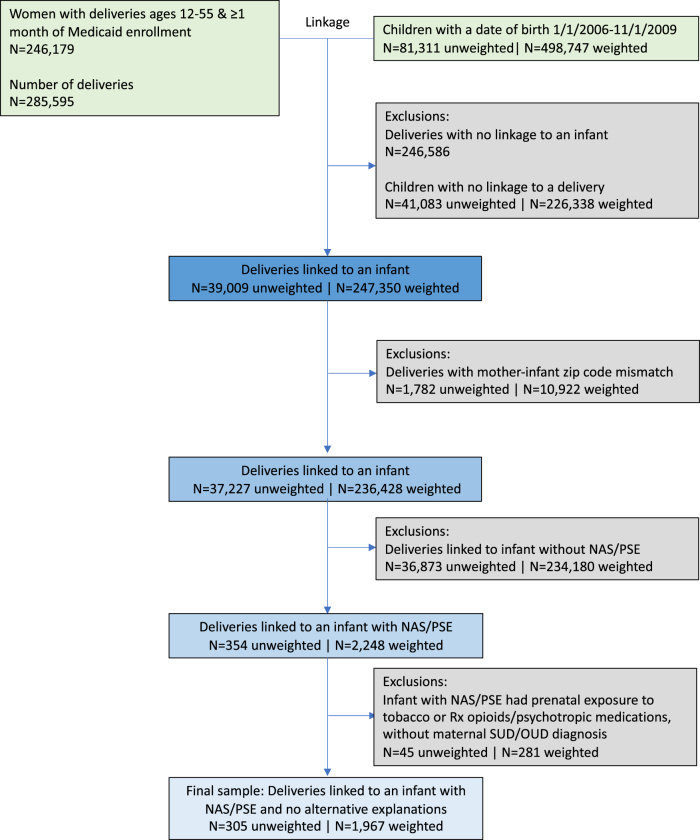
Construction of the sample of mother–infant dyads.

Mothers and infants were identified and linked using previously described methods, briefly described below.^21^ Appendix Figure A1 shows the inclusion and exclusion criteria used to construct the final sample. The initial maternal sample consisted of all women with a claim for a delivery between January 1, 2006 and December 31, 2009. Women were included if they were 12–55 years old, resided in one of the three selected states in the year of delivery, and had at least 1 month of Medicaid enrollment in the 12 months before and including delivery. Deliveries were linked to infants based on family identification numbers and dates of delivery within 5 days of infant birth date. Infants with birth dates after November 1, 2009, were excluded to allow for at least 60 days of postpartum follow-up. Deliveries in which the mother and infant zip code did not match were also excluded. Linked mother–infant dyads were included if the infant had an International Classifications of Disease, Version 9 (ICD-9) code indicating noniatrogenic NAS^22^ and/or PSE within 30 days. To increase the likelihood that we were examining dyads in which the mother had a “true” SUD and thus, a need for SUD treatment postpartum, dyads were excluded if the infant was diagnosed with NAS and the mother had evidence of a potential non-OUD-related cause of NAS, such as prenatal tobacco use, prescription opioids, or psychotropic medications, without a prenatal SUD or OUD diagnosis. As the unit of analysis is the dyad, women with multiple births could be represented more than once in the sample. Appendix Table A1 contains the ICD-9 codes, Healthcare Common Procedure Coding System (HCPCS) codes, and National Drug Codes (NDC) used to develop these inclusion and exclusion criteria.

### Variables

The main outcome variable was maternal receipt of any SUD treatment in the first 60 days postpartum. This time frame was selected to ensure that women had continuous postpartum Medicaid coverage after delivery since before Medicaid expansion under the Affordable Care Act many women with limited resources lost coverage after 60 days due to income-based eligibility restrictions.^23^ Our definition of SUD treatment included both SUD-specific pharmacotherapy (*e.g.*, methadone if covered by Medicaid, buprenorphine) and other behavioral health treatment (*e.g.*, counseling) when accompanied by an SUD or OUD diagnosis on the same claim.

Maternal demographic variables included age, race/ethnicity, rurality of location of residence at time of delivery based on the 2006 National Center for Health Statistics (NCHS) Urban-Rural Classification Scheme for Counties, and state. We grouped counties as follows: large central metropolitan; large fringe metropolitan; and small or medium metropolitan/nonmetropolitan.^24^ Maternal health insurance coverage and health care utilization variables included calendar months of Medicaid enrollment in the 12 months before and including the month of delivery, number of prenatal visits (dichotomized as <7 visits or ≥7 visits, consistent with the literature defining adequate prenatal care when gestational age at delivery is unavailable)^25^ attendance at a postpartum visit (defined as at least one visit for obstetric care between 4 and 60 days after delivery)^25^ and any SUD treatment during the prenatal period. Behavioral health diagnoses included SUD diagnosis in the prenatal period (categorized as any OUD, other SUD without OUD, and no SUD/OUD) and diagnosis of a co-occurring mental health condition (defined as major depression, bipolar disorder, and anxiety disorders, including generalized anxiety and posttraumatic stress disorder, schizophrenia, and perinatal mood disorders) in the prenatal period or 60 days postpartum. Mothers were also considered to have an OUD diagnosis if they had a claim for methadone to treat OUD or a prescription for a buprenorphine formulation indicated for OUD treatment at any point during the third trimester of pregnancy.

Appendix Table A2 lists HCPCS codes, ICD-9 codes, and NDCs used to identify SUD treatment receipt, SUD and OUD diagnoses, prenatal and postpartum visits, and mental health conditions.

### Statistical analysis

Frequencies and percentages describe maternal characteristics of the study sample. Descriptive statistics are reported for women who did and did not receive postpartum SUD treatment, and we compared the distribution of each variable using chi-square tests with a Rao-Scott correction for survey weights. We also plotted the timing of the first SUD treatment received within 60 days of delivery. Analyses were conducted between April 16, 2020, and August 18, 2020, using SAS 9.4 (Cary, NC). This study was approved by the corresponding author's Institutional Review Board with a waiver of consent.

## Results

The final sample included 1967 weighted mother–infant dyads (305 unweighted) in which the infant was diagnosed with NAS and/or PSE in Louisiana, Massachusetts, and Wisconsin from 2006 to 2009. Of these dyads, 15% of mothers received postpartum SUD treatment in the first 60 days following delivery. Nearly half of mothers were 25 years of age or younger, and 68% were white (Table 1). Fewer than half (42%) were diagnosed with SUD or OUD in the prenatal period, and nearly half (46%) had a co-occurring mental health condition in the perinatal period. In 82% of the dyads, the mother was enrolled in Medicaid for at least 7 months in the year before delivery, and about 50% had at least seven prenatal visits. Only 22% attended a postpartum visit in the first 60 days after delivery. Overall, one in five mothers had received any prenatal SUD treatment.

**Table 1.** Sociodemographic Characteristics, Health Insurance Enrollment and Health Care Utilization, and Behavioral Health Conditions Among Mothers of Infants with NAS and/or PSE in Three State Medicaid Programs by Receipt of Postpartum SUD Treatment, 2006–2009

	Total sample^a^	Received any postpartum SUD treatment	Did not receive postpartum SUD treatment	
	*N* (%)	*N* (%)	*N* (%)	
Total	305 unweighted 1967 weighted	58 unweighted 295 weighted (15%)	247 unweighted 1671 weighted (85%)	*p*^b^
Sociodemographic characteristics				
Age				
≤25	49%	35%	51%	0.030
26–34	44%	49%	43%	
≥35	8%	16%	6%	
Race/ethnicity^c^				
White	68%	>87%	65%	<0.001
Black	20%	<3%	24%	
Hispanic	4%	6%	3%	
Other/missing	8%	7%	8%	
Rurality of location of residence				
Large central metro (NCHS 1)	18%	24%	17%	<0.001
Large fringe metro (NCHS 2)	23%	33%	21%	
Medium or small metro/nonmetro (NCHS 3, 4, 5, 6)	51%	22%	56%	
Missing	8%	22%	6%	
State				
Louisiana	43%	7%	49%	<0.001
Massachusetts	32%	35%	32%	
Wisconsin	25%	58%	19%	
Behavioral health conditions				
Type of SUD diagnosed in prenatal period				
Any OUD	25%	71%	17%	<0.001
Other SUD, without OUD	17%	8%	19%	
No SUD/OUD	58%	21%	64%	
Co-occurring mental health condition in prenatal period or 60 days postpartum				
Present	46%	65%	43%	0.008
Not present	54%	35%	57%	
Health insurance enrollment and health care utilization				
Months enrolled in Medicaid in year before delivery^d^				
1–6 months	17%	11%	19%	<0.193
7+ months	82%	89%	81%	
No. of prenatal visits				
<7 visits	51%	52%	51%	0.848
≥7 visits	49%	48%	49%	
Any prenatal SUD treatment				
Yes	20%	68%	12%	<0.001
No	80%	32%	88%	
Attendance at postpartum visit				
Yes	22%	23%	21%	0.862
No	78%	77%	79%	

^a^ The weighted sample of 1967 deliveries corresponds to a sample of 305 unweighted deliveries. Fewer than 11 women had two deliveries during the time period. This table presents percentages of weighted frequencies, which are rounded to the nearest integer and may not sum to 100%.

^b^ Rao-Scott Corrected Chi square tests were performed.

^c^ Race/ethnicity data have been coarsened to protect confidentiality of Medicaid beneficiaries.

^d^ Includes 12 calendar months before and including delivery. Women enrolled 0 months were excluded from analysis.

NAS, neonatal abstinence syndrome; NCHS, National Center for Health Statistics; PSE, prenatal substance exposure; SUD, substance use disorder.

### Comparisons between women who did or did not receive postpartum SUD treatment

Compared with mothers of infants with NAS and/or PSE who did not receive postpartum SUD treatment, mothers who received treatment were older (*p* = 0.03), more likely to be white, and more likely to reside in a large central or fringe metropolitan area (*p* < 0.001 for both comparisons, Table 1). We observed significant differences in SUD treatment by state. Of women who received postpartum SUD treatment, 58% resided in Wisconsin, 35% in Massachusetts, and only 7% in Louisiana. Compared with mothers of infants with NAS and/or PSE who did not receive postpartum SUD treatment, mothers who received treatment were more likely to have an OUD diagnosis (71% vs. 17%), less likely to have another SUD without OUD (8% vs. 19%), and less likely to have no SUD or OUD diagnosis (21% vs. 64%, *p* < 0.001). Those who received postpartum SUD treatment were also more likely to have a co-occurring mental health condition (65% vs. 43%, *p* = 0.008). Of those who received SUD treatment in the postpartum period, 68% had also received any prenatal SUD treatment, while 32% initiated treatment postpartum. In contrast, among women who did not receive postpartum SUD treatment, 12% had received prenatal SUD treatment but did not continue in the first 60 days postpartum, and 88% did not receive any treatment either prenatally or postpartum (*p* < 0.001). There was no statistically significant association between postpartum SUD treatment receipt and months of Medicaid enrollment in the year before delivery, number of prenatal visits, or postpartum visit attendance.

### Timing of first SUD treatment in 60 days after delivery

Among the 15% of women who received postpartum SUD treatment, approximately half (54%) received treatment in the first 2 weeks after delivery, nearly one-third (31%) first received postpartum treatment between 2 and 4 weeks after delivery, and 14%, or about one in seven women, first received treatment >4 weeks postpartum.

## Discussion

In this study of nearly 2000 Medicaid-enrolled linked mother–infant dyads in Louisiana, Massachusetts, and Wisconsin with an infant diagnosed with NAS or PSE, we found that the vast majority of women who likely need SUD treatment did not receive it within 60 days postpartum. Among those who received postpartum SUD treatment, most had received treatment in the prenatal period.

Few studies have examined linked mother–infant dyads affected by substances using Medicaid MAX data.^10,12^ Linking mothers to their infants allowed us to observe that despite 82% of women having 7 or more months of enrollment in the year prior delivery, and about half having at least seven prenatal visits, fewer than half (44%) of women whose infants had NAS and/or PSE had a documented SUD or OUD in the prenatal period. Therefore, studies that rely only on maternal claims data may miss the majority of SUD diagnoses during pregnancy. Our findings suggest that despite women having multiple months of Medicaid coverage prenatally and interacting fairly frequently with the health care system, their likely substance use was often missed. Other studies have found that pregnant and postpartum women with SUD are underrecognized and therefore go undertreated,^6^ and it is well known that untreated SUDs lead to numerous adverse outcomes for mother–infant dyads.^26^ Underdiagnosis is one potential explanation for the small proportion of women with SUD receiving postpartum treatment and highlights a missed opportunity to improve interconception care for those who go on to have another pregnancy.

Our finding that only 15% of women received any SUD treatment, including both pharmacotherapy and nonpharmacologic interventions, in the 60 days postpartum is strikingly low given that their infants were recognized at birth as having had PSE. Although our data are from an earlier period of the opioid crisis, our findings serve as a useful point of reference for more recent analyses and are consistent with other literature covering an overlapping time frame (2007–2014), in which <10% of parenting women with SUD received past year SUD treatment.^27^ These results were particularly striking in Louisiana, which did not provide Medicaid coverage for methadone. Several studies have documented the multiple barriers to care during the perinatal period for women with SUD,^8,28–30^ including stigma, lack of treatment capacity, challenges with daily treatment attendance, and logistical hurdles such as childcare and transportation.^10,31^ These more recent studies documenting multiple barriers suggest that the low rates documented in our study may very well continue to exist and highlight the continued need for programmatic and policy interventions to address barriers to engaging women with SUD in person-centered care during both the prenatal period as well as in the critical months following delivery,^32^ (the “fourth trimester”),^33^ when relapse rates and overdose risk increase.^6,34^

Overall, only one in five women with infants affected by substance exposure attended a postpartum visit within 60 days of delivery, which is consistent with a 2019 study of women with OUD in a single state.^35^ Contrary to our expectations, there was no significant difference between receipt of postpartum SUD treatment and attendance at the postpartum visit. This lack of an association is concerning; ideally, the postpartum visit should be an opportunity to ensure appropriate linkage to SUD treatment during this vulnerable period as well as to address prevalent co-occurring mental health conditions that, consistent with other literature,^10,36–38^ were common in our study sample. It was also unexpected that months of Medicaid enrollment and number of prenatal visits were not associated with postpartum SUD treatment. We suspect that other patient-, provider-, and system-level factors are driving whether women whose infants are identified as exposed to *in utero* substances receive postpartum SUD treatment. Further qualitative research is warranted to examine these factors.

Pregnancy and the postpartum period are often seen as a motivating time for women to newly engage in SUD treatment,^39^ but our data suggest a gap in effectively linking women to counseling or pharmacotherapy among those who were not already engaged before delivery. Specifically, about two-thirds of women who received any postpartum SUD treatment had also received treatment in the prenatal period, and among those who did not receive postpartum SUD treatment, the vast majority (88%) also did not receive SUD treatment prenatally. Taken together, these findings provide further evidence of the importance of engaging women with SUD in the prenatal period and maintaining continuity of care for the chronic disease of addiction throughout the life course of women of reproductive age.^40,41^ Reassuringly, about half of women who received postpartum SUD treatment did so in the first 2 weeks postpartum, and 85% received treatment in the first month postpartum. The finding that most women receiving SUD treatment received it early in the postpartum period, in many cases before the follow-up postpartum visit would have occurred, highlights the potentially valuable role of delivery hospital-based services linking mothers to SUD treatment.

This study had several limitations. First, as with all observational studies, ascertainment of key study variables such as NAS, SUD, or treatment episodes could be subject to misclassification. We sought to mitigate this limitation to some extent by using a broad definition of SUD treatment to capture as many potential treatment episodes as possible, for example, considering mothers to have an OUD diagnosis if they had a claim for methadone to treat OUD or a buprenorphine formulation indicated for OUD treatment during the third trimester. However, we were not able to identify receipt of methadone among women for whom a claim was not submitted, such as those who paid cash or were not continuously enrolled in Medicaid. Another limitation is that postpartum visits or SUD treatment occurring after 60 days postdelivery are not captured in our data. We also do not know the precise *in utero* substance exposures from these data. Finally, our data were limited to analyzing births in three states from 2006 to 2009. Two of these states (Massachusetts and Wisconsin) included methadone in the Medicaid benefit during this time period, which was associated with increased use of MOUD among pregnant women in a prior study.^42^ We do not know if our findings would generalize to other states and years or non-Medicaid populations. However, the examination of linked dyads is a strength, and as noted in our discussion, recent literature continues to describe persistent barriers to SUD treatment in the perinatal period. We therefore believe that our findings are relevant to current policy discussions around reducing inequities in maternal morbidity and mortality as they provide an important baseline for evaluations of policies aimed at improving maternal–infant outcomes.

### Implications for practice and policy

Our finding that among Medicaid-insured women in three states whose infants are recognized as having *in utero* substance exposure, only 15% received any postpartum SUD treatment with counseling and/or pharmacotherapy highlights an important opportunity to better serve mothers with SUD and their infants. This study, using data from the period when the opioid crisis was taking hold across the United States, serves as a useful baseline and context for other recent studies that have documented numerous challenges pregnant women with SUD experience with access to evidence-based care. Given the persistence of these intractable challenges, implementation and evaluation of innovative approaches to overcome them are critically needed. The recent passage of the American Rescue Plan Act of 2021, giving states the option to extend postpartum Medicaid eligibility to 1 year; improving coverage continuity in the period around childbirth for individuals insured by Medicaid^43^ and academic-community initiatives that focus on supporting postpartum women^44^ are promising strategies. Others include efforts to expand provider capacity to manage addiction in pregnancy through telementoring initiatives (*e.g.*, Project ECHO, or Extension for Community Healthcare Outcomes)^45^ and interventions to strengthen rural perinatal care for women with OUD.^46^

## Conclusions

Multipronged approaches at patient, provider, programmatic, and policy levels are needed to overcome the persistent structural barriers to delivering evidence-based SUD treatment in the postpartum period.

## Abbreviations Used

HCPCS: Health care Common Procedure Coding System
ICD-9: International Classifications of Disease, Version 9
MAX: Medicaid Analytic Extract
NAS: neonatal abstinence syndrome
NCHS: National Center for Health Statistics
NDC: National Drug Codes
OUD: opioid use disorder
PSE: prenatal substance exposure
SUD: substance use disorder
ECHO: Extension for Community Healthcare Outcomes

## Appendix

**Table:** Appendix Table A1. Codes Used to Construct the Sample

Diagnosis	Coding system	Codes
NAS and prenatal substance exposure		
NAS	ICD-9	779.5
Iatrogenic NAS exclusions	ICD-9	777.6, 770.7, 779.7, 772.1X, 777.5X, 854.0X, 854.1X
Prenatal substance exposure	ICD-9	760.71, 760.72, 760.75, 760.70, 760.79
Criterion	Coding system	Codes or drug classes
Maternal exclusion criteria		
Tobacco use	ICD-9	305.1X, 649.0X, 989.84, V15.82
Tobacco use	CPT	99406, 99407
Tobacco use	HCPCS	S9075, S9453
Prescription fill for medications that can cause NAS	NDC	*Opioid analgesics (excluding buprenorphine),*^a^*antianxiety medications,*^b^*antidepressants,*^c^*and mood stabilizers*^d^

^a^ NDC listed as opioids and not listed as buprenorphine from the Centers from Disease Control https://www.cdc.gov/drugoverdose/resources/data.html or Medi-Span Electronic Drug File, Version 2 https://www.wolterskluwercdi.com/drug-data/medi-span-electronic-drug-file.

^b^ NDC listed as benzodiazepines from the Centers from Disease Control https://www.cdc.gov/drugoverdose/resources/data.html or as Antianxiety Agents or Hypnotic/Sedatives/Sleep Disorder Agents from Medi-Span Electronic Drug File, Version 2 https://www.wolterskluwercdi.com/drug-data/medi-span-electronic-drug-file.

^c^ NDC listed as antidepressants from HEDIS https://www.ncqa.org/hedis/measures/antidepressant-medication-management/ or Medi-Span Electronic Drug File, Version 2 https://www.wolterskluwercdi.com/drug-data/medi-span-electronic-drug-file.

^d^ NDC listed as antipsychotics from HEDIS https://www.ncqa.org/hedis/reports-and-research/national-collaborative-for-innovation-in-quality-measurement/hedis-measures-for-the-safe-judicious-use-of-antipsychotic-medications-in-children-and-adolescents/ or as Anticonvulsants or Lithium containing compounds from Medi-Span Electronic Drug File, Version 2 https://www.wolterskluwercdi.com/drug-data/medi-span-electronic-drug-file.

CPT, Common Procedural Terminology; HCPCS, Healthcare Common Procedure Coding System; HEDIS, Healthcare Effectiveness Data and Information Set; ICD-9, International Classification of Diseases, Version 9; NAS, neonatal abstinence syndrome; NDC, National Drug Code.

**Table:** Appendix Table A2. Codes Used to Define Variables

Variable	Coding system	Codes or drugclasses
SUD treatment	HCPCS	H0015, S9475, H2036, H2035, S0201, H0005, T1006
SUD treatment	NDC	Buprenorphine^a^
Other behavioral health treatment^b^	CPT	90862, 90804, 90805, 90807, 90808, 90809, 90810, 90811, 90812, 90813, 90814, 90815, 90816, 90817, 90818, 90819, 90821, 90822, 90823, 90824, 90826, 90827, 90828, 90829, 90875, 90876, 90846, 90847, 90849, 90853, 90857, 90806, 90845, 90870, 90871, 90880, 96152, 99510, 96153, 96154, 96155, 99354, 99355
Other behavioral health treatment^b^	HCPCS	J3490, J2315, J8499, H0034, H2010, H0033, M0064, T1502, S9480, S9485, H0035, T2034, H2012, G0410, G0411, H0004, H2032, G0176, H0017, H0018, H0019, T2048
OUD	ICD-9	304.00-304.03; 304.70-304.73, 305.50-305.53, 965.00, 965.01, 965.02, 962.09, E850.0, E935.0
OUD^c^	CPT	H0020, J1230, S0109, J0592, H0034, H2010, H0033, M0064, T1502, 90862
SUD	ICD-9	291.X, 303.X-305.X, 648.30-648.33, 655.50, V6542, 790.3, 980.0, E8600, E8601, E9809, E8552, E9804, E8551, E9801, E8541, E8542, E8532, E8538, E8539, E9803, 9770, 9711, E8543, E8554, E8588, E8508, E95804, 968.5, 967.0-968.3, E851-E852, 969.6-969.7, 969.3-969.5, 969.8-969.9, 970.1, 970.81, 970.89, 965.8-965.9, 357.5, 425.5, 535.3, 571.X
Prenatal visit	ICD-9	V22.X, V23.X
Postpartum visit	ICD-9	V24.1, V24.2
Co-occurring mental health conditions	ICD-9	295.X, 296.X, 311, 648.4X, 300.0X, 309.81

^a^ NDC listed as buprenorphine from the Centers from Disease Control https://www.cdc.gov/drugoverdose/resources/data.html or Medi-Span Electronic Drug File, Version 2 https://www.wolterskluwercdi.com/drug-data/medi-span-electronic-drug-file.

^b^ Other Behavioral Health Treatment codes were considered to indicate SUD treatment if they occurred on the same claim as a SUD or OUD diagnosis.

^c^ Methadone for OUD or buprenorphine injection, if present during third trimester.

OUD, opioid use disorder; SUD, substance use disorder.
